# Reliability of the Peer-Review Process for Adverse Event Rating

**DOI:** 10.1371/journal.pone.0041239

**Published:** 2012-07-26

**Authors:** Alan J. Forster, Monica Taljaard, Carol Bennett, Carl van Walraven

**Affiliations:** 1 The Ottawa Hospital, Ottawa, Ontario, Canada; 2 Department of Medicine, Faculty of Medicine, University of Ottawa, Ottawa, Ontario, Canada; 3 Clinical Epidemiology Program, Ottawa Hospital Research Institute, Ottawa, Ontario, Canada; 4 Institute for Clinical Evaluative Sciences, Ottawa, Ontario, Canada; 5 Department of Epidemiology and Community Medicine, University of Ottawa, Ottawa, Ontario, Canada; University of Maryland, School of Medicine, United States of America

## Abstract

**Background:**

Adverse events are poor patient outcomes caused by medical care. Their identification requires the peer-review of poor outcomes, which may be unreliable. Combining physician ratings might improve the accuracy of adverse event classification.

**Objective:**

To evaluate the variation in peer-reviewer ratings of adverse outcomes; determine the impact of this variation on estimates of reviewer accuracy; and determine the number of reviewers who judge an adverse event occurred that is required to ensure that the true probability of an adverse event exceeded 50%, 75% or 95%.

**Methods:**

Thirty physicians rated 319 case reports giving details of poor patient outcomes following hospital discharge. They rated whether medical management caused the outcome using a six-point ordinal scale. We conducted latent class analyses to estimate the prevalence of adverse events as well as the sensitivity and specificity of each reviewer. We used this model and Bayesian calculations to determine the probability that an adverse event truly occurred to each patient as function of their number of positive ratings.

**Results:**

The overall median score on the 6-point ordinal scale was 3 (IQR 2,4) but the individual rater median score ranged from a minimum of 1 (in four reviewers) to a maximum median score of 5. The overall percentage of cases rated as an adverse event was 39.7% (3798/9570). The median kappa for all pair-wise combinations of the 30 reviewers was 0.26 (IQR 0.16, 0.42; Min = −0.07, Max = 0.62). Reviewer sensitivity and specificity for adverse event classification ranged from 0.06 to 0.93 and 0.50 to 0.98, respectively. The estimated prevalence of adverse events using a latent class model with a common sensitivity and specificity for all reviewers (0.64 and 0.83 respectively) was 47.6%. For patients to have a 95% chance of truly having an adverse event, at least 3 of 3 reviewers are required to deem the outcome an adverse event.

**Conclusion:**

Adverse event classification is unreliable. To be certain that a case truly represents an adverse event, there needs to be agreement among multiple reviewers.

## Introduction

Several influential studies of adverse events have focused public attention on health care safety. Adverse events are defined as poor patient outcomes caused by medical care. Peer-review of adverse outcomes is required to determine whether an adverse event occurred. Since peer review can be unreliable, it is possible that previous estimates of adverse event prevalence are inaccurate.

The peer review process can be considered a diagnostic test in which the physician’s decision about whether or not an adverse event occurred is the test result. Using this paradigm, one can define a peer reviewer’s accuracy using standard test characteristics such as sensitivity, specificity, and likelihood ratios. The latter statistic can be combined with adverse event prevalence to estimate the probability of an adverse event given a particular adverse event rating.

The principal obstacle to measuring peer-review accuracy is the absence of a gold-standard for adverse events.[1]–[5] A gold standard test correctly distinguishes cases from non-cases and permits the easy calculation of both adverse event prevalence and physician accuracy. [6] However, even in the absence of a gold-standard, adverse event prevalence and reviewer accuracy can be estimated using latent class analysis.[7]–[11] We recently published a simulation study in which we used latent class analysis to demonstrate the impact of adverse event prevalence and the accuracy of adverse event judgments on the probability that adverse events truly occurred. [5] We demonstrated that: using one reviewer to determine adverse event status is inadequate; combining three physician ratings increased the reliability of determining adverse event status; and, the probability that an adverse event truly occurred (as a function of peer-reviewer ratings) was dependent on the accuracy and variability of those ratings.

In this study, we evaluated the variability of a larger number of peer-reviewers of adverse outcomes. This work let us better understand the range of variability in physician reviewer ratings and determine the impact that this variability has on estimates of reviewer accuracy. Finally, it will also let us determine the number of reviewers who judge that an adverse event occurred required for the probability that an adverse event truly occurred to reach certain thresholds.

## Methods

### 2.1 Study Overview

We asked 30 physicians to review a set of 319 case reports describing poor outcomes experienced by medical and surgical patients after discharge from hospital. Physicians assessed each case summary and rated the extent to which they felt the poor outcome was attributable to healthcare management. We analyzed these ratings to determine the impact of unreliable peer reviews to develop strategies for improving its accuracy. The study was approved by the Ottawa Hospital Research Ethics Board.

### 2.2 Physician Peer Reviewers

We recruited a convenience sample of 30 American or Canadian board-certified physicians to review 319 adverse event case reports. To identify potential reviewers, we asked editorial board members of the Journal of Hospital Medicine to advertise within their respective institutions. We also advertised the project and need for reviewers to investigators who participated in the Canadian Adverse Event Study. Physicians received standardized training similar to that used by Brennan et al. and Baker et al. [2], [12] We asked participating physicians to complete a short survey to define basic demographic details, experiences with respect to the peer-review process and prior litigation, and opinions about patient safety. All physicians provided written informed consent to participate in the study and signed confidentiality forms.

### 2.3 Patient Population

The Outcomes After the Hospitalization (OAtH) Study was a multi-centre prospective cohort study that measured the association of continuity of care with patient outcomes after discharge from hospital. It was conducted at 11 teaching and community hospitals in Ontario, Canada. [13] Consenting medical and surgical patients received standardized telephone follow-up interviews at one, three, and six months post discharge to determine functional status, health service utilization, and the occurrence of pre-specified clinical outcomes (including the development of new symptoms, emergency room visits, hospitalizations, and death). Overall, the OAtH study recruited 5035 patients with 85.8% completing follow-up at all three time points.

### 2.4 Generation of Case Reports

When OAtH patients experienced a pre-specified outcome (any healthcare visit in the 6 month follow-up period in which the patient experienced a new problem or exacerbation of an existing problem), standard information was collected (from the patient or their surrogate) including: the event date; the patient’s response to the outcome; the healthcare system’s responses to, and interventions for, the outcome; whether the outcome was resolved after the interventions; and what the patient was told was the cause of the outcome. For patients who returned to a hospital we obtained and reviewed the ED record of treatment and the discharge summary for the return visit. A case report form that described the patient’s diagnoses, hospital care, other treatments, outcomes and resolution, was generated for each adverse outcome that we identified. Overall, 2669 outcomes occurred in 1592 patients. For this study, we randomly selected 319 outcomes involving 216 patients. All 30 physicians reviewed the 319 outcomes.

### 2.5 Physician Ratings

The 30 physicians used a six-point ordinal scale to rate the cause of each adverse outcome. This scale has been used in all previous major adverse event studies.[14]–[19] The discrete levels of this scale were defined as: 1– no evidence for management causation; 2– slight evidence for management causation; 3– management causation less than 50–50 but close call; 4– management causation more than 50–50 but close call; 5– strong evidence for management causation; 6– virtually certain evidence for management causation. As per convention in the majority of adverse event studies, all outcomes rated by physicians as a 4 or higher on this scale were classified as an adverse event by that peer-reviewer.

To perform the reviews, we used a web-based application developed specifically for this study. The reviewer accessed each case report form over a secured internet connection. In addition, we removed all identifying personal health information from each form.

### 2.6 Analysis

We first summarized the results of the physician peer reviews. To do so, we calculated the median and inter-quartile range of the ordinal response ratings for each clinician. We also calculated the number and percent of cases rated as AEs by physician and by case. 95% confidence intervals around proportions were calculated using the (large-sample) normal approximation to the binomial distribution.

To explore the effect of physician characteristics on reviewer ratings, we used the 6-point rating as a continuous outcome variable in a mixed-effects linear regression model. The physician predictors obtained from the survey – including country of practice, years since graduation from medical school, previous experience performing peer-review, previous civil litigation experience and attitude toward patient safety (as measured by level of agreement with the statement: “Medical errors are a major quality problem in health care”) - were specified as fixed effects. To account for clustering of repeated ratings by the same physician, we specified the physician as a random effect in the model. We first evaluated the association of the 6-point rating with each variable individually by including them individually in the model. We then evaluated multivariable associations by including all predictors in the model, and eliminating predictors in a stepwise fashion until all predictors remaining in the model were significant at α = 0.10.

In all further analyses, we dichotomized adverse event ratings in which scores of 4, 5 or 6 were classified as an adverse event. We used three indices to measure the level of agreement between physician reviewers for the dichotomised rating. First, we computed all pair-wise agreement between raters using simple kappa statistics and calculated the median and inter-quartile range of these statistics for each physician. Second, we calculated an overall kappa statistic for multiple raters using the methods described by Fleiss. [20].

Finally, we conducted latent class analyses with two latent classes (i.e. adverse event present or absent) to estimate the true prevalence of adverse events along with the sensitivity and specificity of each peer reviewer. Latent class analysis is a well-developed statistical methodology, which can be used to address a key limitation in adverse event detection, namely the absence of a gold standard classification. The term “latent class” refers to the fact that the true adverse event status of each patient is not observed. With latent class analysis, the observed data, namely multiple independent ratings of the same case by a panel of reviewers, are used to estimate the unobserved prevalence of adverse events, reviewer sensitivity, and reviewer specificity using the method of maximum likelihood estimation. The maximum likelihood procedure selects the parameter estimates that best fit the patterns of agreement and disagreement observed among reviewers. [21] Maximum likelihood estimates of our model parameters were computed by means of the Expectation Maximization (EM) algorithm. Using the approach described by Uebersax and Grove for a fixed panel of reviewers, [21] the latent class analysis was conducted by allowing sensitivities and specificities to vary among individual reviewers.

In order to obtain the posterior probability of an adverse event for all possible numbers of positive ratings (out of 30 raters), we repeated the latent class analysis assuming a common sensitivity and specificity for all reviewers. This was done using a straightforward application of Bayes’ rule as presented in Uebersax and Grove [21] (Appendix S1). Finally, we varied the maximum number of raters from k = 2 to k = 30 and repeated the above calculations using Bayes’ rule to determine the posterior probability of an adverse event for all possible number of positive ratings. For each number of raters (i.e. between 2 and 30), we determined the minimum number of physician ratings indicating an adverse event for the posterior probability of an AE classification to exceed 50%, 75% and 95%.

Latent class analyses were conducted using LEM. [22] All other analyses were conducted using SAS v9.1 (SAS institute Inc., Cary, NC, USA).

## Results

### 3.1 Description of the Reviewers

Table 1 describes the demographic characteristics of the 30 physician reviewers. The majority of the reviewers were American (80%) with a median time since graduation from medical school of nine years (range: 4–42). The majority (66.7%) had previous chart auditing experience and few reviewers (16.7%) had previously been involved in civil litigation. All reviewers felt that medical errors are a major quality problem in health care.

**Table 1:** Description of the reviewers.

Characteristic	N = 30
**Country of practice**	
Canada	6 (20%)
US	24 (80%)
**Years since Medical School graduation** *	9 (4, 42)
**Previous chart auditing experience**	
Yes	20 (66.7%)
Years of experience*	3 (0.1, 30)
No	10 (33.3%)
**Previous civil litigation involvement**	
Yes	5 (16.7%)
No	25 83.3%)
**“Medical errors are a major quality problem in health care”**	
Agree	19 (63.3%)
Agree somewhat	11 (36.7%)
Neutral	0
Disagree somewhat	0
Disagree	0

* Median (range).

### 3.2 Observed Adverse Event Ratings

Table 2 summarizes adverse event ratings for the 319 cases by all 30 reviewers. The overall median score on the 6-point ordinal scale was 3 (IQR 2,4) but the median individual rater score ranged from a minimum of 1 (in four reviewers) to a maximum median score of 5 (in one reviewer). With the dichotomised scale (score of 4 or above indicating an adverse event; score of 3 or less indicating no adverse event), 3798 of 9570 ratings (39.7%) were classified as an adverse event. Of the 319 cases, 111 (34.8%, 95% CI 30.0% to 40.0%) received a median score of at least 4 and were therefore rated as an adverse event by a majority of reviewers. Seven cases were unanimously rated as due to the underlying disease (i.e. *not* an adverse event). No case was unanimously rated as an adverse event but one case was rated as such by 28 of 30 reviewers.

**Table 2:** Summary of the 30 individual peer-reviewers of the 319 cases.

Reviewer number*	Median score on six point ordinal scale† (IQR)	Number of cases rated as an adverse event‡N (%)	Model-based reviewer operating characteristic	
			Sensitivity	Specificity
1	1 (1,2)	22 (7)	0.0562	0.92
2	1 (1,2)	43 (13)	0.2703	0.9824
3	1 (1,3)	51 (16)	0.25	0.9181
4	1 (1,4)	90 (28)	0.5542	0.9532
5	2 (1,3)	55 (17)	0.3524	0.9832
6	2 (1,3)	76 (24)	0.3784	0.883
7	2 (1,4)	84 (26)	0.3992	0.8542
8	2 (1,4)	86 (27)	0.3129	0.7678
9	2 (1,4)	96 (30)	0.4952	0.8671
10	2 (1,5)	103 (32)	0.527	0.8537
11	2 (1,5)	125 (39)	0.7717	0.9366
12	2 (1,5)	134 (42)	0.8347	0.9386
13	2 (1,5)	137 (43)	0.8159	0.9048
14	2 (1,6)	135 (42)	0.794	0.8975
15	3 (1,4)	129 (40)	0.6847	0.838
15	3 (1,5)	130 (41)	0.6964	0.8424
17	3 (1,5)	156 (49)	0.7858	0.7676
18	3 (1,6)	155 (49)	0.8404	0.8207
19	3 (2,4)	93 (29)	0.5815	0.9593
20	3 (2,4)	115 (36)	0.4909	0.7523
21	3 (2,5)	154 (48)	0.7665	0.7627
22	4 (1,5)	162 (51)	0.8889	0.8217
23	4 (1,5)	167 (52)	0.875	0.7804
24	4 (1,5)	171 (54)	0.8485	0.7342
25	4 (2,5)	160 (50)	0.8484	0.7984
26	4 (2,5)	176 (55)	0.891	0.7417
27	4 (2,5)	181 (57)	0.9025	0.7224
28	4 (3,4)	190 (60)	0.706	0.4999
29	4 (3,5)	204 (64)	0.9314	0.613
30	5 (2,5)	218 (68)	0.9104	0.513
**Overall**	**3 (2,4)**	**3798 (40)**	**0.65**	**0.82**

* Physician reviewers were numbered based on their median 6-point scale score ranking.

† 1– Definitely due to disease, 6– definitely due to medical care.

‡ Ordinal scale dichotomized: 1–3 ‘Due to disease’; 4–6 ‘Due to medical care’.

§ Area under the receiver operating characteristic curve.

### 3.3 Physician Factors Influencing Reviewers’ Ratings

The bivariable regression analyses revealed no statistically significant associations between the rating score and physician factors including country of practice (p = 0.99), years since graduation from medical school (p = 0.51), previous experience performing peer review (p = 0.17), years of experience (p = 0.45), previous civil litigation experience (p = 0.48), or attitude toward patient safety (p = 0.65). Likewise, none of the physician-level predictors were significant in the multivariable regression analyses.

### 3.4 Agreement between Physician Reviewers

**Figure 1:**
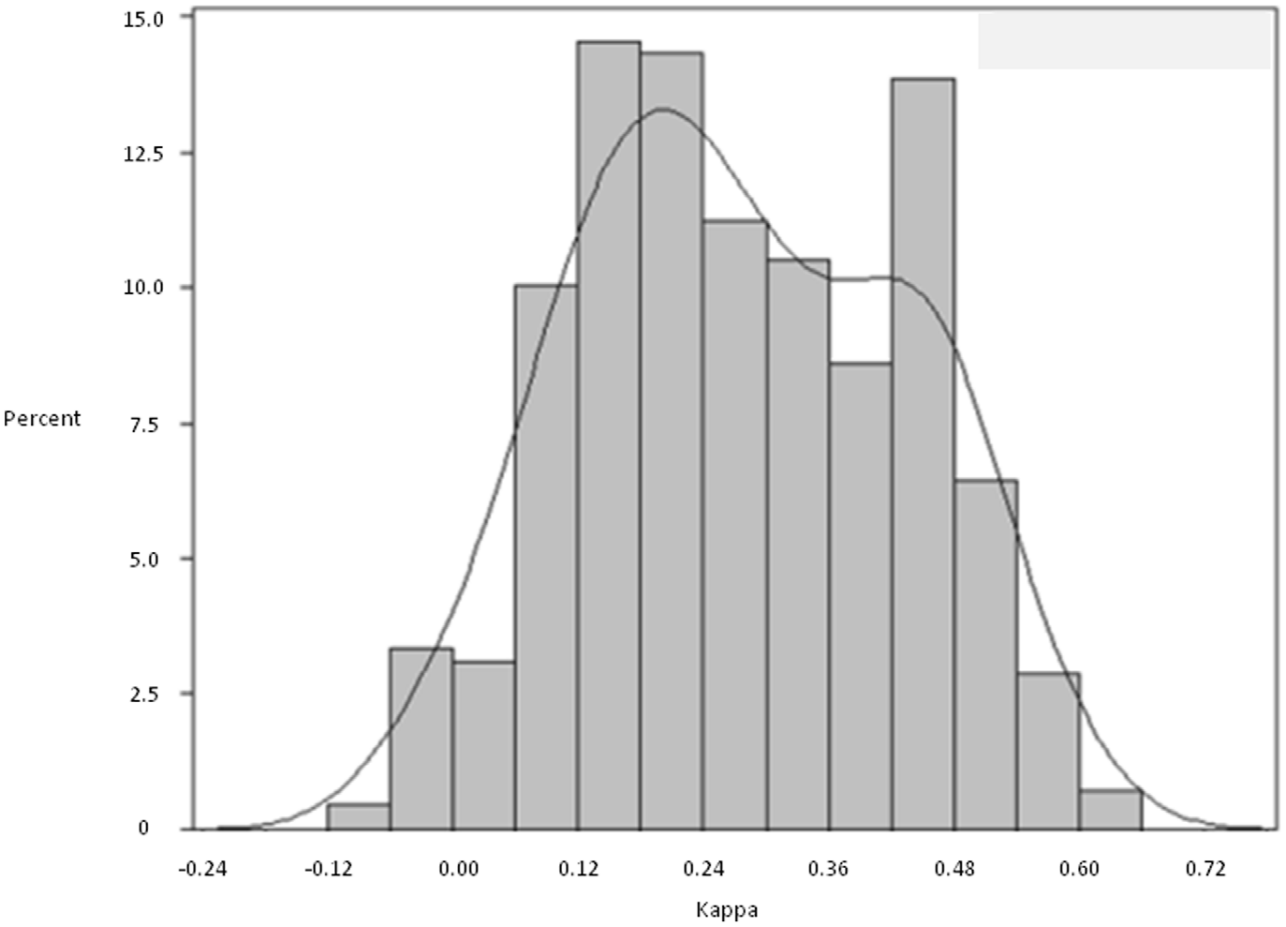
Kappa scores for 30 reviewer pairs. This plot presents the distribution of the kappa statistics for all possible pairwise comparisons between the 30 reviewers for all 319 cases.

Both the simple kappa statistic (calculated for all possible combinations of pairs of reviewers) and the overall kappa statistic (calculated across all 30 reviewers) indicated only fair agreement. The median kappa for all pair-wise combinations of the 30 reviewers was 0.26 (IQR 0.16 to 0.42; Range −0.07 to 0.62). Figure 1 presents the summary frequency distribution of pairwise kappa statistics for all possible combinations of reviewers; it shows a bimodal distribution of agreement levels with peaks at kappa statistics 0.20 and 0.45. The overall kappa statistic accounting for all the reviewers simultaneously was 0.28.

### 3.5 Estimating Adverse Event Prevalence and Reviewer Test Characteristics

The estimated prevalence of adverse events from the latent class analysis was 46.4% (95% CI 40.8% to 51.9%). The model-based sensitivities and specificities for each reviewer are presented in Table 2: reviewer sensitivity ranged from 0.056 to 0.931 (mean 0.65); reviewer specificity ranged from 0.500 to 0.983 (mean 0.82). The estimated prevalence of adverse events using the common sensitivity model was similar to that which allowed reviewer sensitivity and specificity to vary: 46.4%, 95% CI 40.8% to 51.9%); the estimated common sensitivity was 0.64 (95% CI 0.626 to 0.660); and the estimated common specificity was 0.83 (95% CI 0.813 to 0.839).

Table 3 presents the probability of an adverse event by the number of reviewers (out of 30) who rated the case as an adverse event. As these results were generated from the model assuming common sensitivity and specificity, they should be interpreted with respect to an “average” or randomly selected reviewer. The probability that the case truly was an adverse event remained less than 1% if nine or fewer of the 30 reviewers rated it as an adverse event. This probability increased dramatically as more reviewers rated the case as adverse event. When at least 14 of 30 reviewers rated the case as an adverse event, the probability that an adverse event truly occurred exceeded 99%.

**Table 3:** Posterior probability of event or non-event given the number of positive ratings.

Total Number of Adverse Event ratings out of 30 raters	Probability that case truly is an Adverse Event
0 (0%)	0.0000
1 (3.3%)	0.0000
2 (6.6%)	0.0000
3 (9.9%)	0.0000
4 (13.2%)	0.0000
5 (16.5%)	0.0000
6 (20.0%)	0.0000
7 (23.3%)	0.0000
8 (26.7%)	0.0003
9 (30.0%)	0.0026
10 (33.3%)	0.0219
11 (36.7%)	0.1612
12 (40.0%)	0.6225
13 (43.3%)	0.9340
14 (46.7%)	0.9918
15 (50.0%)	0.9990
16 (53.3%)	0.9999
17 (56.7%)	1.0000
18 (60.0%)	1.0000
19 (63.3%)	1.0000
20 (66.7%)	1.0000
21 (70.0%)	1.0000
22 (73.3%)	1.0000
23 (76.7%)	1.0000
24 (80.0%)	1.0000
25 (83.3%)	1.0000
26 (86.7%)	1.0000
27 (90.0%)	1.0000
28 (93.3%)	1.0000
29 (96.7%)	1.0000
30 (100%)	1.0000

These estimates are from the latent class model that assumed a common sensitivity and specificity for all reviewers.

### 3.6 Determining the Number of Reviewers Required to Meet Posterior Probability Thresholds

Table 4 lists the minimum number of ratings of an adverse event required for the probability that a case is truly an adverse event exceeds 50%, 75%, and 95%. This table informs decisions regarding the number of reviewers one would have to recruit to obtain a threshold of certainty of adverse event occurrence (assuming a reviewer with “average” operating characteristics). For example, if one wished there to be at least 95% certainty of an adverse event, then one would need a minimum of three reviewers. If one reviewer dissented in their review, then one would have to recruit a fourth reviewer to have the potential to reach the certainty threshold or acknowledge there was uncertainty over a particular case’s classification.

**Table 4:** Number of adverse event ratings required for true probability of adverse event to exceed 50%, 75%, and 95%.

	Number of cases that need to be rated as an adverse event required for true probability of an adverse event		
Number of Raters Per Case	≥50%	≥75%	≥95%
2	1	2	Not possible
3	2	2	3
4	2	3	3
5	2	3	4
6	3	3	4
7	3	4	5
8	4	4	5
9	4	5	5
10	4	5	6
11	5	5	6
12	5	6	7
13	6	6	7
14	6	7	7
15	6	7	8

This table enables the identification of the number of raters required to obtain a given certainty that a case ‘truly’ represents an adverse event. For example, if an investigator wished to be 95% certain a case represented an adverse event, then it would be necessary to have a minimum of three reviewers and all three would need to agree. With only two reviewers, once could be at best 75% certain a case represented an adverse event.

## Discussion

The most impressive finding from this study of outcome reviews for a group of 30 physicians was the high degree of variation in their ratings. This variation was highlighted by the finding that not one case was rated as an adverse event by all 30 reviewers. While these physicians varied extensively by experience, training, and attitudes towards patient safety, none of these factors were significantly associated with adverse event ratings. Our pair-wise comparisons and the results of our latent class analysis demonstrated tremendous variation in reviewer agreement and accuracy, respectively. Despite the variations in reviewer behaviour, reasonable posterior probabilities for adverse event occurrence could be achieved through the combination of reasonable numbers of reviewers.

Ideally, explicit criteria would be used to identify adverse events. However, as others have noted, [2]–[4], [23], [24] the complex nature of medical care prohibits the creation of explicit criteria for the vast majority of adverse event types, especially given the infinite combinations of patient characteristics, treatments and outcomes. In the absence of explicit criteria, implicit case review is required to identify adverse events. Our results show that it is necessary to have several reviewers agree before assigning the cause of an outcome in a particular situation.

Currently, no standards exist for determining consensus in implicit reviews or who should be involved. Our results show that there are large variations in agreement between providers. Some pairs of reviewers have high levels of agreement, whereas others rarely agree. The bimodal nature of the Figure 1 likely results because of distinct populations of reviewers – those that agree versus those that do not. We are not aware of this being demonstrated previously.

Because agreement defines accuracy, there is a need to consider it when selecting reviewers for an adverse event study. It should be possible to calibrate physician ratings against a training set in order to select a physician to participate. Therefore, in the selection of participants for a consensus review process, it is necessary to consider the reviewer’s accuracy, which is a function of their likelihood of considering a case to be due to medical care and their likelihood of agreeing with his/her colleagues. Our results show that it might be possible to define a desired level of reviewer accuracy, which would result in improved reliability and reduce the requirements of multiple reviews.

Furthermore, one could generate an algorithm to combine the ratings of multiple reviewers. For example, assume an investigator desired greater than 95% certainty that a case represented an adverse event. Assuming the reviewer accuracy we identified is generalizable, it would be necessary to recruit at least three reviewers per case and cases could only be classified as an adverse event if all three agreed.

Prior studies of adverse events have shown much greater agreement amongst reviewers than we have. [2], [4], [17], [18], [25] This could be a due to reviewer selection. While it might be appealing to consider using our results to revise estimates of adverse event prevalence from these studies, we do not recommend doing so. We were interested in evaluating the review process, so recruited physicians from different institutions and different types of training. The reviewers were also geographically dispersed and did not have a chance to communicate or calibrate their reviews. Irrespective of reasons for the difference between our findings and those of prior adverse event studies, there are several implications worth considering. Thus, we did not select reviewers of similar background and training. High agreement amongst reviewers in the published safety studies is most likely due to the recruitment of similarly accurate reviewers. This would lead to an improved performance in terms of the number of reviewers required to define a reasonable posterior probability of an event being ‘true’. However, if one were to assume a similar distribution of reviewer accuracy that we found then the likelihood that prior studies led to overestimates is very high.

Our observed variability in reviewer accuracy might reflect a non-sensical approach to defining adverse events. In our study, as in all of the major patient safety studies, we considered adverse events as an “all-or-none” phenomenon. Although this approach is consistent with the current methods to classify cases, it may not be the correct way to approach this issue. It is likely that in reality medical care contributes to outcome in varying degrees. For example, a wound infection is much more likely in patients undergoing dirty versus clean surgery. Therefore, care could be considered to contribute relatively more to wound infections in patients undergoing clean surgeries than in patients undergoing dirty surgeries. The variation in clinicians’ ratings may partially reflect the clinical reality that poor outcomes are multi-factorial and rarely ‘black-and-white’. To account for the variable amount that medical care could contribute to outcomes, a different modelling approach could be employed which did not consider adverse events as an all or none phenomenon. This should be the focus of future work.

In summary, this study has three important implications for adverse event studies. First, given variability in reviewer accuracy, any attempt to measure adverse events will require an approach combining input from multiple independent reviewers. Second, the number of reviewers required to reach agreement depends on the accuracy of reviewers and estimated prevalence of adverse events. Third, the variability in reviews might indicate an incorrect conceptual model that considers treatment related harm as an all or none phenomenon. Future work should focus on developing models to improve our ability to account for reviewers’ opinions of the variable contributions care plays in determining adverse outcomes.

## Supporting Information

Appendix S1 Example of posterior probability calculation.
(DOCX)
