# Supplementary material for: Reliability of the Peer-Review Process for Adverse Event Rating
Source: PLoS One. 2012 Jul 26;7(7):e41239. doi: 10.1371/journal.pone.0041239 (PMC3406022; doi:10.1371/journal.pone.0041239)
Supplement: Appendix S1 — Example of posterior probability calculation. (DOCX) [file pone.0041239.s001.docx]

**Appendix S1**: Example of posterior probability calculation.

Suppose we want to calculate the posterior probability of an adverse event, given that 14 out of 30 reviewers assigned a positive rating:

$$P\left(AE|14\text{ out of 30 positive ratings}\right)$$

We calculate this probability using a straightforward application of Bayes’ rule:

$$P\left(A|B\right)=\frac{P(A\cap B)}{P\left(B\right)}=\frac{P(B|A)P(A)}{P(B|A)P(A)+P(B|\bar{A})P(\bar{A})}$$

and the binomial formula:

$$P(X=x)=\left(\begin{matrix} n \\ x \end{matrix}\right)p^{x}\left(1-p\right)^{n-x}$$

From the latent class analysis, the prevalence of an adverse event is , common sensitivity is $p=sens=P(\text{Positive rating|AE)=0.6432}$ and

1-specificity=$P(\text{Positive rating|}\overline{\text{AE}}\text{)}$=1-0.8264=0.1736.

Thus, using Bayes’ rule:

$$\begin{matrix} P\left(AE|14\text{ out of 30 positive ratings}\right) \\ =\frac{P(AE)\times P\left(14\text{ out of 30 positive ratings|AE}\right)}{P(AE)\times P\left(14\text{ out of 30 positive ratings|AE}\right)+P(\overline{AE})\times P\left(14\text{ out of 30 positive ratings|}\overline{\text{AE}}\right)} \end{matrix}$$

First calculate the numerator using the binomial formula:

$$\begin{matrix} P\left(14\text{ out of 30 positive ratings|AE}\right)=\left(\begin{matrix} 30 \\ 14 \end{matrix}\right)sens^{14}\left(1-sens\right)^{30-14} \\ =_{30}C_{14}\times0.6432^{14}\times\left(1-0.6432\right)^{16} \\ =0.02081 \end{matrix}$$

Then calculate the denominator:

$$\begin{matrix} P(AE)\times P\left(14\text{ out of 30 positive ratings|AE}\right)+P(\overline{AE})\times P\left(14\text{ out of 30 positive ratings|}\overline{\text{AE}}\right) \\ =0.4755\times0.02081+\left(1-0.4755\right)\times\left(\begin{matrix} 30 \\ 14 \end{matrix}\right)(1-spec)^{14}\left[1-\left(1-spec\right)\right]^{30-14} \\ =0.009895+(1-0.4755)\left[_{30}C_{14}\times0.1736^{14}\times\left(1-0.1736\right)^{16}\right] \\ =0.009895+0.0000812 \\ =0.00998 \end{matrix}$$

This yields the result:

$$P\left(AE|14\text{ out of 30 positive ratings}\right)=\frac{0.4755\times0.02081}{0.00998}=0.9919$$
